# Multi-institutional randomized clinical study on the comparative effects of intracavital chemotherapy alone versus immunotherapy alone versus immunochemotherapy for malignant effusion

**DOI:** 10.1038/sj.bjc.6690421

**Published:** 1999-05

**Authors:** Y Nio, H Nagami, K Tamura, M Tsubono, M Nio, M Sato, K Kawabata, H Hayashi, T Shiraishi, S Imai, T Tsuchitani, J Mizuta, M Nakagawa, M Fukumoto

**Affiliations:** First Department of Surgery, Shimane Medical University, Shimane, Izumo, 693-8501, Japan; Sato Hospital, Osaka, Hirakata, 573-1141, Japan; Shin-Kawabata Hospital, Kyoto, Nagaokakyo, 617-0825, Japan; Department of Surgery, Tango Central Hospital, Kyoto, Mineyama, 627-0012, Japan; First Department of Surgery, Kobe Central Hospital, Hyogo, Kobe, 650-0046, Japan; Department of Surgery, Kishiwada Municipal Hospital, Osaka, Kishiwada, 596-0042, Japan; Department of Internal Medicine, Eiko Hospital, Kagawa, Takuma, 769-1100, Japan; Department of Surgery, Shimane Prefectural Central Hospital, Shimane, Izumo, 693-8555, Japan; First Department of Pathology, Faculty of Medicine, Kyoto University, Kyoto, Japan

**Keywords:** malignant effusion, chemotherapy, immunotherapy, OK-432 (Picibanil), intracavital therapy

## Abstract

The current prospective randomized study was designed to compare the effects of intracavitary (i.c.) chemotherapy vs immunotherapy vs immunochemotherapy for malignant effusion. Between 1992 and 1995, a total of 42 patients with malignant effusion were registered, and 41 patients were eligible for statistical analysis. The primary diseases of the eligible patients included 27 gastric, four colorectal, four pancreatic, three lung, two liver and one oesophageal cancers. The patients with malignant effusion were randomly assigned into one of three i.c. therapeutic regimens: chemotherapy alone with weekly injection of anticancer agents (ACAs: cisplatin, mitomycin-C, adriamycin, etc.) (Group A, *n* = 13); immunotherapy alone with weekly injection of streptococcal preparation OK-432 (Group B, *n* = 14); or immunochemotherapy with ACAs and OK-432 (Group C, *n* = 14). The response of the effusion, patient survival and the kinetics of cytokines in the effusion were compared. There were no differences in the patients' backgrounds. The side-effects of the regimens included pain, anorexia, fever, leucopenia and anaemia and there were no differences in their incidence among the three groups. One patient died after cisplatin (CDDP) administration in Group A. Cytologic examination revealed that tumour cells in the effusion disappeared in 23% of Group A cases, 36% of Group B cases and 36% of Group C cases. The malignant effusion did not disappear in any of the Group A cases; however, the effusion disappeared in 29% of Group B cases and 43% of Group C cases (*P* = 0.03, Group A vs Group C). Furthermore, the 50% survival period was 1.6 months for Group A, 2.4 months for Group B and 3.5 months for Group C. The 6-month survival rate was 7% for Group A, 6% for Group B and 34% for Group C, and the 1-year survival rate was 0%, 0% and 17% respectively (*P* = 0.048, Group A vs Group C by the log-rank test). The analysis of the cytokine kinetics revealed a prominent increase in the level of interleukin-6 in the effusion in Group C. These results suggest that i.c. immunochemotherapy with OK-432 and ACAs may be more beneficial than i.c. chemotherapy alone or immunotherapy alone. © 1999 Cancer Research Campaign


British Journal of Cancer (1999) **80**(5/6), 775–785
© 1999 Cancer Research Campaign
Article no. bjoc.1998.0421

### Multi-institutional randomized clinical study on the comparative effects of intracavital chemotherapy alone versus immunotherapy alone versus immunochemotherapy for malignant effusion

**Y Nio[1], H Nagami[1], K Tamura[1], M Tsubono[2], M Nio[2], M Sato[2], K Kawabata[3], H Hayashi[3], T Shiraishi[4], S Imai[5], T Tsuchitani[6], J Mizuta[7], M Nakagawa[8] and M Fukumoto[9]**

[1]First Department of Surgery, Shimane Medical University, 89-1 Enya-cho, Izumo, Shimane 693-8501, Japan; [2]Sato Hospital, Hirakata, Osaka 573-1141, Japan; [3]Shin-Kawabata Hospital, Nagaokakyo, Kyoto 617-0825, Japan; [4]Department of Surgery, Tango Central Hospital, Mineyama, Kyoto 627-0012, Japan; [5]First Department of Surgery, Kobe Central Hospital, Kobe, Hyogo 650-0046, Japan; [6]Department of Surgery, Kishiwada Municipal Hospital, Kishiwada, Osaka 596-0042, Japan; [7]Department of Internal Medicine, Eiko Hospital, Takuma, Kagawa 769-1100, Japan; [8]Department of Surgery, Shimane Prefectural Central Hospital, Izumo, Shimane 693-8555, Japan; [9]First Department of Pathology, Faculty of Medicine, Kyoto University, Kyoto, Japan

**Summary** The current prospective randomized study was designed to compare the effects of intracavitary (i.c.) chemotherapy vs immunotherapy vs immunochemotherapy for malignant effusion. Between 1992 and 1995, a total of 42 patients with malignant effusion were registered, and 41 patients were eligible for statistical analysis. The primary diseases of the eligible patients included 27 gastric, four colorectal, four pancreatic, three lung, two liver and one oesophageal cancers. The patients with malignant effusion were randomly assigned into one of three i.c. therapeutic regimens: chemotherapy alone with weekly injection of anticancer agents (ACAs: cisplatin, mitomycin-C, adriamycin, etc.) (Group A, $n = 13$); immunotherapy alone with weekly injection of streptococcal preparation OK-432 (Group B, $n = 14$); or immunochemotherapy with ACAs and OK-432 (Group C, $n = 14$). The response of the effusion, patient survival and the kinetics of cytokines in the effusion were compared. There were no differences in the patients' backgrounds. The side-effects of the regimens included pain, anorexia, fever, leucopenia and anaemia and there were no differences in their incidence among the three groups. One patient died after cisplatin (CDDP) administration in Group A. Cytologic examination revealed that tumour cells in the effusion disappeared in 23% of Group A cases, 36% of Group B cases and 36% of Group C cases. The malignant effusion did not disappear in any of the Group A cases; however, the effusion disappeared in 29% of Group B cases and 43% of Group C cases ($P = 0.03$, Group A vs Group C). Furthermore, the 50% survival period was 1.6 months for Group A, 2.4 months for Group B and 3.5 months for Group C. The 6-month survival rate was 7% for Group A, 6% for Group B and 34% for Group C, and the 1-year survival rate was 0%, 0% and 17% respectively ($P = 0.048$, Group A vs Group C by the log-rank test). The analysis of the cytokine kinetics revealed a prominent increase in the level of interleukin-6 in the effusion in Group C. These results suggest that i.c. immunochemotherapy with OK-432 and ACAs may be more beneficial than i.c. chemotherapy alone or immunotherapy alone.

**Keywords:** malignant effusion; chemotherapy; immunotherapy; OK-432 (Picibanil); intracavital therapy

Malignant effusion appears in the terminal stage of cancers, especially in digestive organ cancer, lung cancer and breast cancer. Despite the recent progress in multidisciplinary treatment employing chemotherapy, radiotherapy, immunotherapy, hyperthermia and other therapies, the most effective therapy for malignant effusion has not been established and the prognosis of patients with malignant effusion is very poor.

The intracavitary (i.c.) administration of anticancer agents (ACAs) has been applied for the treatment of malignant effusion; however, most patients usually die within 3 months after initiation of the therapy. In general, i.c. chemotherapy with bleomycin (BLM) or cisplatin (CDDP), has been applied. Paladine et al reported that i.c. BLM for malignant pleural effusion from lung cancer resulted in a 63% response with a mean survival period of 41 days for responders; however, they indicated that various malignancies showed different responses, and effusion from breast and ovarian cancers responded well to i.c. chemotherapy (Paladine et al, 1976). Osterowski (1986) reported that intrapleural (i.pl.) BLM resulted in an 80.5% response for malignant pleural effusion with a 1-year disease-free rate of 19%. He also reported that breast carcinoma responded well to i.pl. BLM: for malignant ascites, intraperitoneal (i.p.) BLM resulted in a 62.5% response with a 1-year survival rate of 6.25%. Rusch et al (1991) reported that i.pl. CDDP and cytosine arabinoside (AraC) for patients with malignant pleural effusion from various malignancies resulted in 39% response, and a median survival of 5.7 months. These reports suggest that: (1) the response of effusions differs among the primary diseases and effusion from breast carcinoma seems to respond better than those from other malignancies; and (2) peritoneal effusion is more resistant to i.c. chemotherapy than pleural effusion. However, with respect to the effects of i.c. chemotherapy on effusions from digestive organ cancers, there are

*Received 14 January 1998*
*Revised 1 November 1998*
*Accepted 25 November 1998*

*Correspondence to:* Y Nio very few reports from the USA and European countries. Paladine reported that effusions from colonic and gastric cancers were resistant to i.c. chemotherapy and all patients died within 3 months (Paladine et al, 1976).

Recently, various new agents have been introduced for the treatment of malignant effusion. Among them, i.c. immunotherapy with streptococcal preparation, OK-432, has been reported to be very beneficial in controlling malignant effusion (Katano and Torisu, 1982; Inoue et al, 1993; Torisu et al, 1983; Kawagoe and Masuda, 1986). OK-432 is a preparation of *Streptococcus pyogenes*, type A3, and this agent has been used as an anti-tumour immunomodulator for gastric, lung and other cancers (Kimura et al, 1976; Tokai Cooperative Study Group for Adjuvant Chemoimmunotherapy of Stomach Cancer, 1981; Kim, 1987; Watanabe and Iwa, 1987; Maehara et al, 1992). The i.c. or intralesional injection of OK-432 is reported to be effective even when administered alone (Torisu et al, 1983; Fukuma et al, 1986; Fujita, 1987); however, intralesional injection of OK-432 also seems to be beneficial in conjunction with other adjuvant therapies, including radiotherapy (Mukai et al, 1995). This suggests that the effects of i.c. OK-432 may also be augmented when combined with other therapies, especially chemotherapy. As far as we know, there has been only one report on the comparative effects of i.c. OK-432 and chemotherapy, and it is also reported that i.c. OK-432 for malignant effusion caused by lung cancer achieved a higher complete response rate (73%) than i.c. mitomycin C (MMC) (41%); however, there was no significant difference in the median survival of patients (Luh et al, 1992). There have been no reports comparing i.c. OK-432 alone and combination therapies with OK-432 and chemotherapy.

The present study was designed to compare the effects of OK-432 alone versus chemotherapy alone versus immunochemotherapy with OK-432 and ACAs.

### MATERIALS AND METHODS

### Registration and randomization of the patients

This clinical study was carried out by the Shimane–Kyoto Research Group for Cancer Immunochemotherapy, and was open to patients from August 1992.

Survival was the true end point. We planned for 15 eligible patients per group to ensure that the trial would have a power (1-β) of 0.80 to detect a ratio (2.5) of the median survival for Group C to the median survival for Group A or B based on a clinical report (Torisu et al, 1983) with a significance level (a) of 0.05 (George and Desu, 1974; Gehan, 1975).

Several basic criteria had to be met before patients were included in the study: (1) cytological proof of malignant effusion; (2) digestive organ cancer or lung cancer as the primary disease; (3) performance status < 3 (ECOG scale); (4) progressive malignant effusion, which was measurable by physical examination (abdominal size) or by X-ray, echography, or computerized tomography (CT).

Contraindications to patient selection included: (1) total disability (PS = 4, ECOG score); (2) prior chemotherapy, radiotherapy or immunotherapy within 4 weeks; (3) an active infectious disease; (4) severe anaemia (haemoglobin < 9.0 g $dl^{-1}$), leucopenia (< 3000 $mm^{-3}$), thrombocytopenia (< 70 000 $mm^{-3}$), azotemia (creatinine > 2.0 mg $dl^{-1}$), or liver dysfunction (GOT, GPT, alkaline phosphatase levels > four-fold normal limits); (5) an allergy to penicillin (OK-432 contains penicillin); (6) severe heart disease or a concomitant malignant disease; and (7) pregnancy. All patients and their families were fully informed with regard to the experimental nature of the treatment programme, and informed consent was obtained.

After registration, the patients were randomly assigned to three groups: Group A – chemotherapy alone, Group B – immunotherapy alone and Group C – immunochemotherapy, according to a random number table communicated using a telephone- or fax-based centre-call method. The registration centre was located at the First Department of Surgery, Shimane Medical University. The study was supervised by Dr Manabu Fukumoto, (Associate Professor, First Department of Pathology, Kyoto University) and the protocol was reviewed by the Ethics Committee consisting of Dr Norimichi Kan (Rakuyo Hospital, Kyoto), Dr Kazuhisa Ohgaki (Kyoto Police Hospital, Kyoto) and Dr Hiroshi Kodama (Kodama Breast Clinic, Kyoto).

### Treatment protocol

Under the universal coverage of the Japanese health insurance system, which was instituted in 1961, ACAs available for clinical use are specified strictly according to their indications by the Japanese Ministry of Health and Welfare. We have therefore classified the ACAs and the primary tumours causing the pleural effusion and ascites in this study according to their indications, when creating the protocol. The treatment protocol is summarized in Table 1. Group A patients were treated with i.c. chemotherapy alone with weekly injections of ACAs; ACAs included cisplatin (CDDP; Briplatin, Bristol Myers Japan, Tokyo, Japan; or Randa, Nippon Kayaku, Tokyo, Japan), carboplatin (CBDCA; Bristol Myers Japan), mitomycin-C (MMC; Kyowa-Hakko Co. Ltd, Tokyo, Japan), adriamycin (ADR; Kyowa-Hakko), and farmorubicin (EPIR; Kyowa-Hakko). Group B patients received i.c. immunotherapy alone with weekly injection of streptococcal preparation, OK-432. Group C patients were treated with i.c. immunochemotherapy with ACAs and OK-432. The dosage of the administered agents are summarized in Table 1. ACAs were diluted in 20–50 ml of distilled water or physiological saline, and administered directly into the peritoneal or pleural cavity usually

**Table 1** Therapy protocol

| |
|---|
| Group A (ACA group) |
| The following ACAs were injected weekly into the peritoneal or pleural cavity, singly or in combination: |
| CDDP at 30 mg $m^{-2}$ (or CBDCA at 300 mg $m^{-2}$) |
| MMC at 6 mg $m^{-2}$ |
| ADR at 30 mg $m^{-2}$ (or EPIR at 30 mg $m^{-2}$) |
| Group B (OK-432 group) |
| OK-432 at 1~10 KE was injected weekly into the peritoneal or pleural cavity |
| Group C (ACA and OK-432 group) |
| One cycle of the regimen included intraperitoneal or intrapleural injections of ACA on day 1 and OK-432 on day 4; the injections were repeated weekly |

The regimens were continued as long as possible. If intracavital injection became impossible due to the disappearance or decrease of effusion, the patients received systemic (intravenous or intramuscular) administration of the agents. ACA, anticancer agent; CDDP, cisplatin; CBDCA, carboplatin; MMC, mitomycin-C; ADR, adriamycin; EPIR, 4′-epirubicin; KE, Klinische Einheit (one KE is equivalent to 0.1 mg of dried cocci).

 © Cancer Research Campaign 1999

through a silicon catheter, which was inserted into the peritoneal or pleural cavity under guidance with an echogram. OK-432 is a penicillin-treated, lyophilized preparation of *Streptococcus pyogenes*, group A type 3 (Picibanil, Chugai Pharmaceutical Co. Ltd, Tokyo, Japan). The Klinische Einheit (KE) is used to express the dosage of the preparation; 1 KE of OK-432 contains 0.1 mg of the dried cocci (Sakurai et al, 1972). OK-432 was also administered alone or in conjunction with ACAs via a silicon catheter directly into the peritoneal or pleural cavity. Intracavital injections of OK-432 were started at a dose of 1–5 KE weekly, and the dose was gradually increased to 5–10 KE as a maintenance dose according to the patient's condition. For patients with a good performance status, drainage was not performed to avoid hypoproteinaemia or dehydration as well as to induce tumour cells and effusion-associated lymphocytes to react with ACAs and/or OK-432. If the patients complained of dyspnoea or other serious effusion-derived symptoms, the effusion was drained until the symptoms disappeared. According to the advice of the Ethics Committee, if the malignant effusion disappeared and the agents could not be injected into the cavity, the ACAs were administered intravenously (i.v.), or 5-fluorouracil (5-Fu) or its derivatives (UFT or 5′-deoxy-fluorouridine) were given orally (p.o.) as a maintenance chemotherapy for patients in Group A and Group C, and OK-432 was injected subcutaneously (s.c.) or intramuscularly (i.m.) at 1–5 KE weekly for patients in Group B and Group C. The duration of treatment with s.c. or i.m. administration of OK-432 was decided by the doctors with informed consent obtained if treatment was discontinued.

The examinations of haematology, serum biochemistry, serum tumour markers and evaluation of symptomatic and performance status were routinely performed at weekly intervals, sometimes more frequently. The results of the therapy were assessed every 4 weeks by measurement of abdominal size, X-ray, echogram, CT, etc. If the disease appeared to progress, the patients were either managed symptomatically and supportively or offered alternative experimental regimens if their general condition seemed to be appropriate.

### Evaluation of the response of the effusion to the therapies

The objective response of the malignant effusion was assessed using chest X-ray, CT, or echography with the following criteria: (1) a complete response (CR) indicated a total disappearance of effusion for at least 4 weeks; (2) a partial response (PR) was defined as 50% or greater reduction in effusion compared to the original effusion volume for at least 4 weeks; (3) no change (NC) indicated less than a 50% reduction in effusion and/or not greater than a 25% increase in effusion compared to the original volume; and (4) progressive disease (PD) was defined as greater than a 25% increase of the original effusion volume. The response was initially evaluated by the investigator at the participating institution, and then re-evaluated by the supervisor. The duration of response was measured from the first day of injection of the agents to the day of increase in the effusion.

### Evaluation of the kinetics of cytokines in the effusion before and after therapy

The kinetics of cytokines in the malignant effusion were assessed. The cytokines, including tumour necrosis factor alpha (TNF-$\alpha$), interleukin-2 (IL-2), interferon gamma (IFN-$\gamma$) and interleukin-6 (IL-6), were measured at Otsuka Tokyo Assay Laboratories Co. Ltd, Tokyo, Japan.

### Evaluation of side-effects

WHO standard criteria for toxicity (Miller et al, 1981) were used. The ileus is a well-known side-effect of OK-432 when it is administered into the peritoneal cavity. This OK-432-associated ileus is caused by adhesion and is different from paralytic ileus, which is usually caused by other ACAs. The OK-432-associated ileus cannot be classified according to the WHO standard criteria, and the grade of ileus caused by OK-432 was classified according to our original criteria for bowel obstruction: grade 0, no symptoms; grade 1, nausea but no vomiting; grade 2, intermittent obstruction but no intervention and no parenteral nutrition; grade 3, intermittent obstruction, no intervention, but requires parenteral nutrition; grade 4, complete obstruction, requires intervention and parenteral nutrition.

### Patient follow-up

All the patients were followed up by physical examination, general X-ray examination, ultrasonography (US), CT, routine haematologic and biochemical examinations, and serum tumour marker assays.

### Statistical evaluation

The effects of the therapies were evaluated with respect to the response rate of the malignant effusion and the survival rate after therapy. Chi-square and Mann–Whitney *U*-tests were used to compare the backgrounds of patients in the three groups. The overall survival was calculated by the Kaplan–Meier method (Kaplan and Meier, 1958). A statistical comparison of the survival rates among the three groups was made by the generalized Wilcoxon test (Gehan, 1965) and the log-rank test (Peto et al, 1977). A *P*-value of less than 0.05 was considered to be significant. The statistical analyses were carried out using SAS computer software.

### RESULTS

### Patient entry and exclusion for evaluation

Patient entry was stopped in July 1995 because differences in the survival rates of Group A and Group C were significant. Between 1992 and 1995, a total of 42 patients with malignant effusion were registered: 41 patients (97.6%) were eligible for statistical analysis, and one patient of Group B was excluded due to misregistration (the patient was treated with a different immunomodulator). The primary diseases of the eligible patients included 27 gastric, four colorectal, four pancreatic, three lung, two liver and one oesophageal cancers. Group A included 13 patients, Group B included 14 patients and Group C included 14 patients. The background factors of the eligible patients are summarized in Table 2 and there were no differences in the patients' backgrounds.

### Drug administration and toxicity

The administered agents and the doses are summarized in Table 3. Three kinds of ACAs were administered in Group A: CDDP in

© Cancer Research Campaign 1999

**Table 2** Background factors

| | Protocol | | |
|---|---|---|---|
| | A (*n* = 13) | B (*n* = 14) | C (*n* = 14) |
| Sex | | | |
| Male | 8 | 7 | 10 |
| Female | 5 | 7 | 4 |
| Age | | | |
| < 60 | 5 | 2 | 6 |
| ≥ 60 | 8 | 12 | 8 |
| Primary disease | 7 | 7 | 4 |
| Recurrent disease | 6 | 7 | 10 |
| Effusion | | | |
| Pleural effusion | 3 | 4 | 1 |
| Ascites | 10 | 10 | 11 |
| Both | 0 | 0 | 2 |
| Origin | | | |
| Oesophageal | 0 | 1 | 0 |
| Gastric | 11 | 7 | 9 |
| Colorectal | 0 | 3 | 1 |
| Pancreatic | 1 | 2 | 1 |
| Hepatocellular | 0 | 0 | 2 |
| Lung | 1 | 1 | 1 |

**Table 3** Dose of the administered agents

| | Protocol | | |
|---|---|---|---|
| | A (*n* = 13) | B (*n* = 14) | C (*n* = 14) |
| Local injection | | | |
| OK-432 (KE) | | 23.9 ± 5.3 (*n* = 14) | 19.2 ± 4.2 (*n* = 14) |
| CDDP (mg) | 94.4 ± 30.8 (*n* = 8) | | 138.9 ± 24.0 (*n* = 9) |
| MMC (mg) | 16.0 ± 3.1 (*n* = 4) | | 9.3 ± 5.5 (*n* = 3) |
| EPIR (mg) | | | 185.0 ± 65.0 (*n* = 2) |
| CBDCA (mg) | 300 (*n* = 1) | | 450 (*n* = 1) |
| 5-FU (mg) | | | 1000 (*n* = 1) |
| Systemic injection | | | |
| OK-432 (i.m.) | | 17.0 ± 14.4 (*n* = 4) | 33.3 ± 12.7 (*n* = 4) |
| CDDP | 115.0 ± 15.0 (*n* = 2) | | 58.3 ± 22.1 (*n* = 3) |
| EPIR | 53.3 ± 13.3 (*n* = 3) | | |
| 5-FU | 3687.5 ± 2035 (*n* = 4) | | 2750 ± 1750 (*n* = 2) |

ACA, anticancer agent; CDDP, cisplatin; CBDCA, carboplatin; MMC, mitomycin-C; ADR, adriamycin; EPIR, 4′-epirubicin; KE, Klinische Einheit (one KE is 0.1 mg of dried cocci); i.m., intramuscularly.

nine patients, MMC in three patients and CBDCA in one patient. The mean dose of OK-432 in Group B was 23.9 ± 5.3 KE. Five kinds of ACAs were administered in Group C patients: CDDP in ten patients, MMC in three patients, EPIR in two patients, CBDCA in one patient and 5-FU in one patient. The patients were administered i.v. or p.o. ACAs and/or i.m. or s.c. OK-432 after a decrease or disappearance of the malignant effusion. The profiles of the systemic therapies are summarized in Table 3.

The toxicities and side effects of the regimens are summarized in Table 4. Toxicity was seen in 46% (6/13) of Group A patients, and side-effects included the leucopenia, thrombocytopenia, fever, pain and anorexia. However, none of these toxicities was serious. In Group B, toxicity of i.c. OK-432 was seen in 29% (4/14) of the patients, and side-effects included fever, anaemia, leucopenia, leucocytosis and anorexia. The incidence of side-effects was highest (57%, 8/14) in Group C, and the toxicities included anaemia, fever, leucopenia and pain. However, in addition to these side-effects, OK-432 injection into the peritoneal cavity resulted in a special problem: two patients suffered from adhesion ileus after the complete disappearance of ascites. They both needed total parenteral nutrition even after the disappearance of ascites until their death due to the recurrence of malignant ascites. However, it is very difficult to differentiate this kind of ileus from that due to progression of carcinomatosis.

### Responses of malignant effusion

Cytologic examination revealed that tumour cells disappeared in 23% (3/13) of patients in Group A, 36% (5/14) of patients in Group B and 36% (5/14) of patients in Group C; there were no differences in cytologic effects (Table 5). The malignant effusion shrunk after treatment in 31% (4/13) of patients in Group A, but never disappeared. By contrast, the effusion disappeared in 29% (4/14) of patients in Group B and 43% (6/14) of patients in Group C ($P$ = 0.03, Group A vs Group C (Table 5).

 *© Cancer Research Campaign 1999*

**Table 4** Side-effects[a]

| | Protocol | Incidence (number/total) A (*n* = 13) 6/13 (46%) | B (*n* = 14) 4/14 (36%) | C (*n* = 14) 8/14 (57%) |
|---|---|---|---|---|
| Fever | Overall | 0 | 2 (14%) | 4 (29%) |
| | Grade 1 | 1 | 1 | 2 |
| | 2 | 0 | 1 | 2 |
| | 3 ~ 4 | 0 | 0 | 0 |
| Pain | Overall | 1 (8%) | 0 | 1 (7%) |
| | Grade 1 | 1 | 0 | 0 |
| | 2 | 0 | 0 | 1 |
| | 3 ~ 4 | 0 | 0 | 0 |
| Anorexia | Overall | 1 (8%) | 1 (7%) | 1 (7%) |
| | Grade 1 | 1 | 1 | 0 |
| | 2 | 0 | 0 | 1 |
| | 3 ~ 4 | 0 | 0 | 0 |
| Leucopenia | Overall | 2 (15%) | 0 (0%) | 2 (14%) |
| | Grade 1 | 2 | 0 | 0 |
| | 2 | 0 | 0 | 1 |
| | 3 | 0 | 0 | 0 |
| | 4 | 0 | 0 | 1 |
| Leucocytosis | Overall | 0 | 1 (7%) | 1 (7%) |
| | Grade 1 | 0 | 1 | 1 |
| | 2 ~ 4 | 0 | 0 | 0 |
| Thrombocytopenia | Overall | 2 (15%) | 0 (0%) | 1 (7%) |
| | Grade 1 | 1 | 0 | 0 |
| | 2 ~ 3 | 0 | 0 | 0 |
| | 4 | 1 | 0 | 1 |
| Anaemia | Overall | 1 (8%) | 2 (14%) | 5 (36%) |
| | Grade 1 | 1 | 0 | 0 |
| | 2 | 0 | 1 | 4 |
| | 3 | 0 | 1 | 1 |
| | 4 | 0 | 0 | 0 |
| Bowel obstruction[b] | Overall | 1 (8%) | 1 (7%) | 2 (14%) |
| | Grade 1 ~ 2 | 0 | 0 | 0 |
| | 3 | 1 | 0 | 1 |
| | 4 | 0 | 1 | 1 |
| Death | | 1 (8%)[c] | 0 | 0 |

[a] According to WHO standard criteria for toxicity. [b]Classified as described in Materials and Methods. [c]Due to acute renal failure caused by CDDP administration.

**Table 5** Comparison of therapeutic effects

| | Protocol A (*n* = 13) | B (*n* = 14) | C (*n* = 14) |
|---|---|---|---|
| Response of effusion | | | |
| a. *Objective response*[a] | | | |
| CR | 0 | 4 (29%) | 6 (43%) |
| PR | 4 (31%) | 1 (7%) | 3 (21%) |
| NC | 3 | 4 | 3 |
| PD | 3 | 0 | 0 |
| Unevaluable[b] | 3 | 5 | 2 |
| b. *Cytologic response* | | | |
| Disappearance | 3 (23%) | 5 (36%) | 5 (36%) |
| No change | 5 | 2 | 3 |
| Unevaluable[b] | 5 | 7 | 6 |
| Survival | | | |
| Median survival days | 74 | 51 | 115 |

[a] $P = 0.039$ by $\chi^2$ test. [b]Due to the patients' death within 4 weeks after initiation of the therapy. CR, complete response; PR, partial response; NC, no change; PD, progressive disease.

© Cancer Research Campaign 1999

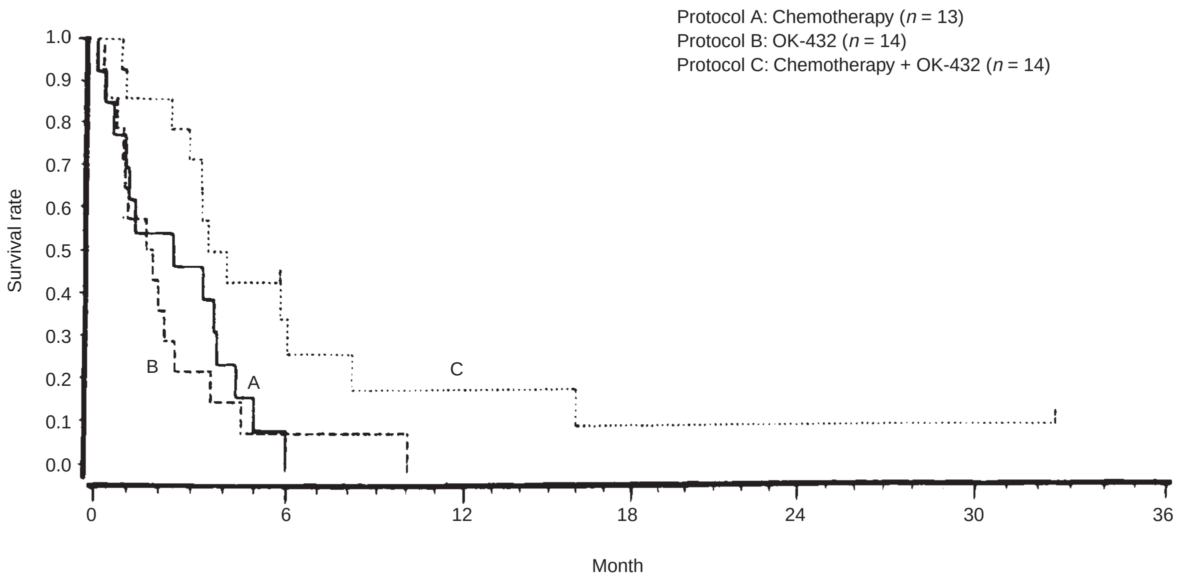

**Figure 1** Survival curves of the three groups. Group A vs Group B: log-rank test; $\chi^2$ = 0.2106, $P$ = 0.6463. Generalized Wilcoxon test; $\chi^2$ = 0.3937, $P$ = 0.5304. Risk ratio (95% CI) = 1.472 (0.606–3.575). Group A vs Group C: log-rank test; $\chi^2$ = 3.8995, $P$ = 0.0483. Generalized Wilcoxon test; $\chi^2$ = 3.0496, $P$ = 0.0808. Risk ratio (95% CI) = 0.652 (0.419–1.014)

### Survival

The 50% survival period was 1.6 months for Group A, 2.4 months for Group B and 3.5 months for Group C. The 6-month survival rate was 7% for Group A, 6% for Group B and 34% for Group C, and the 1-year survival rate was 0%, 0% and 17% respectively ($P$ = 0.048, Group A vs Group C by log-rank test) (Figure 1).

In the present study, two patients survived for more than 1 year after initiation of the therapy, and both patients belonged to Group C. Their clinical courses are summarized in Figures 2 and 3. One patient (45-year-old male) had massive pleural effusion and ascites, which were caused by recurrent hepatocellular carcinoma. He was assigned to Group C and treated with weekly i.p. and i.pl. injections of EPIR (30 mg $m^{-2}$) and OK-432 (1 KE per body). The effusion completely disappeared 6 months later (CR); 3 years have passed and he is still free from effusion, although the recurrent tumour still exists but is in remission (Figure 2A–C). Another patient (85-years-old male) had malignant pleural effusion due to lung cancer (adenocarcinoma) (Figure 3A). He was also assigned to Group C, and was treated with a weekly i.pl. injection of EPIR (10 mg $m^{-2}$) and OK-432 (1 KE per body). He received injections a total of 20 times, and the effusion shrunk (MR), but never disappeared. Fortunately, he lived with a good quality of life for more than 1 year (survival period, 16 months) (Figure 3A–C).

### Cytokines in the effusion

The cytokines in the effusion are summarized in Figure 4. In several patients it was difficult to draw effusion before and after therapy, due to the death of the patient or the disappearance of effusion. Accordingly, the kinetics of cytokines were evaluated in four patients of Group A, three patients of Group B and eight patients of Group C, both before and after therapy. IL-1α and IL-1β were not detected in any of the three groups. The level of IFN-γ did not change in Group A; however, it increased threefold in one patient of Group B and 2.3-fold and 3.3-fold in two patients of Group C, whose effusion disappeared. The level of TNF-α increased in one patient of Group A, did not change in any patient of Group B, and increased in two patients and decreased in two patients of Group C. The level of IL-6 increased in three patients of Group A, one patient of Group B and all patients of Group C.

### DISCUSSION

The present study demonstrated that i.c. OK-432 alone was more effective in controlling the malignant effusion than i.c. chemotherapy alone. Intracavital OK-432 resulted in the disappearance of effusion in 29% of patients; however, i.c. chemotherapy did not completely eliminate the effusion in any of the patients. There were no differences in life-prolonging effects between i.c. chemotherapy and i.c. OK-432, and these therapies produced no 1-year survivors. There is a report on the comparative effects of i.c. OK-432 and chemotherapy which reported that pleurodesis with OK-432 for malignant effusion caused by lung cancer achieved a higher complete response rate (73%) than did pleurodesis with MMC (41%); however, there was no significant difference in the median survival of patients who received the two treatments (5.8 months for OK-432 and 5.1 months for MMC). These results are compatible with the present results.

Intracavital OK-432 is a standard regimen for malignant effusion in Japan. Torisu et al (1983) first reported that i.c. OK-432 was very beneficial in the management of malignant ascites. Intraperitoneal OK-432 resulted in a mean survival time of 10.2 months and a 50% survival time of 9.0 months compared to 3.1 months and 2.6 months for palliative therapy respectively (Torisu et al, 1983). They also reported that neutrophil-mediated tumour cell destruction is one of the mechanisms responsible for the anti-tumour effects of OK-432 (Katano and Torisu, 1982). Uchida and Micksche (1983) reported that the cytotoxicity of effusion-associated lymphocytes (EAL) was activated against allogeneic and autologous tumour cells after the i.pl. administration of OK-432 in humans. Saito et al reported that i.c. OK-432 augmented the LAK activity of

© Cancer Research Campaign 1999

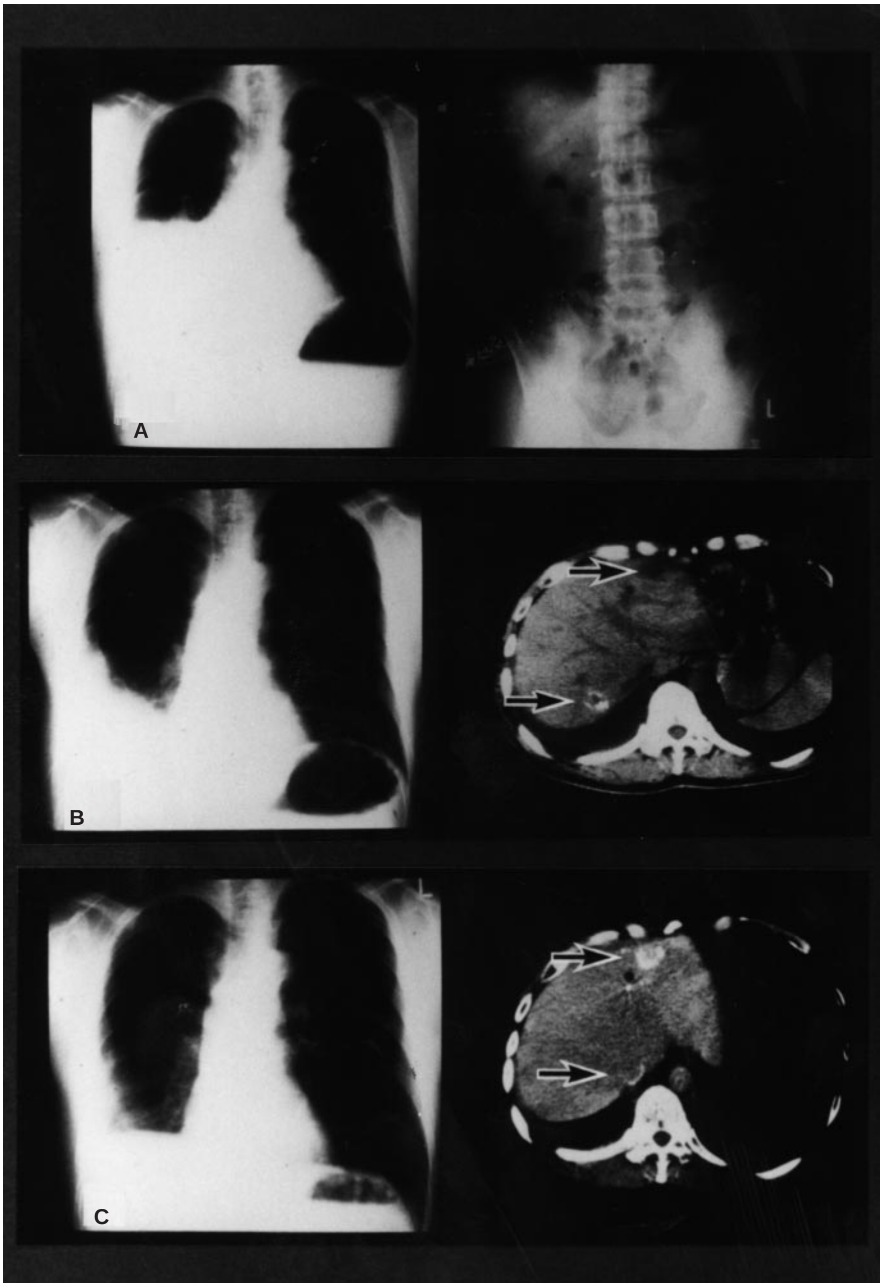

**Figure 2** A 45-year-old male patient with malignant pleural effusion and ascites, which were caused by recurrent hepatoma. The patient had been disease-free for 3 years after right hepatectomy for hepatoma. However, he suddenly suffered from dyspnoea in 1993, and came to our outpatient section. A chest X-ray examination showed massive pleural effusion (**A**), and dyspnoea was relieved after removing about 1000 ml of pleural effusion. Cytological examination revealed malignant cells (hepatoma cells), and an abdominal CT also demonstrated the recurrent hepatomas. He was told that he had malignant effusion in both the pleural and peritoneal cavities, which was caused by the recurrent hepatoma. He then agreed to the treatment according to the protocol. He was registered and assigned to Group C and treated with weekly intraperitoneal and intrapleural injections of EPIR (30 mg $m^{-2}$) and OK-432 (1 KE per body). At first the effusions seemed to be resistant to the regimen; however, the consistent treatment resulted in the disappearance of effusion after 6 months (CR) (**B**). According to his preference, he was treated three times with an intra-arterial injection of lipiodol (2 ml) and EPIR (20 mg) into the hepatic artery. Three years have passed and he is still free from effusion, although the recurrent tumour still exists (**C**)

EAL in mice (Saito et al, 1986). These reports have concentrated on the immunomodulating effects of OK-432; however, it has also been reported that OK-432 showed direct cytotoxic and cytostatic effects on tumour cells (Sakurai et al, 1972; Ono et al, 1973; Nio et al, 1989*b*). In addition, OK-432 modulates the surface molecules on tumour cells. It is reported that i.c. administration of OK-432 to patients with malignant ascites resulted in the enhancement of intra-cellular adhesion molecule (ICAM)-I expression on tumour cells (Kitsuki et al, 1994), and that in vitro treatment of tumour cells with OK-432 resulted in enhanced susceptibility to cytotoxic effector cells (NK cells and LAK cells) (Nio et al, 1990; Mizutani et al, 1992*a*). These results suggest that OK-432 may augment the direct cytotoxicity of EAL against tumour cells as well as the binding of EAL to tumour cells in malignant effusion.

Although the present study had an important problem in that we could not use the same ACAs for all protocols due to restrictions

© Cancer Research Campaign 1999

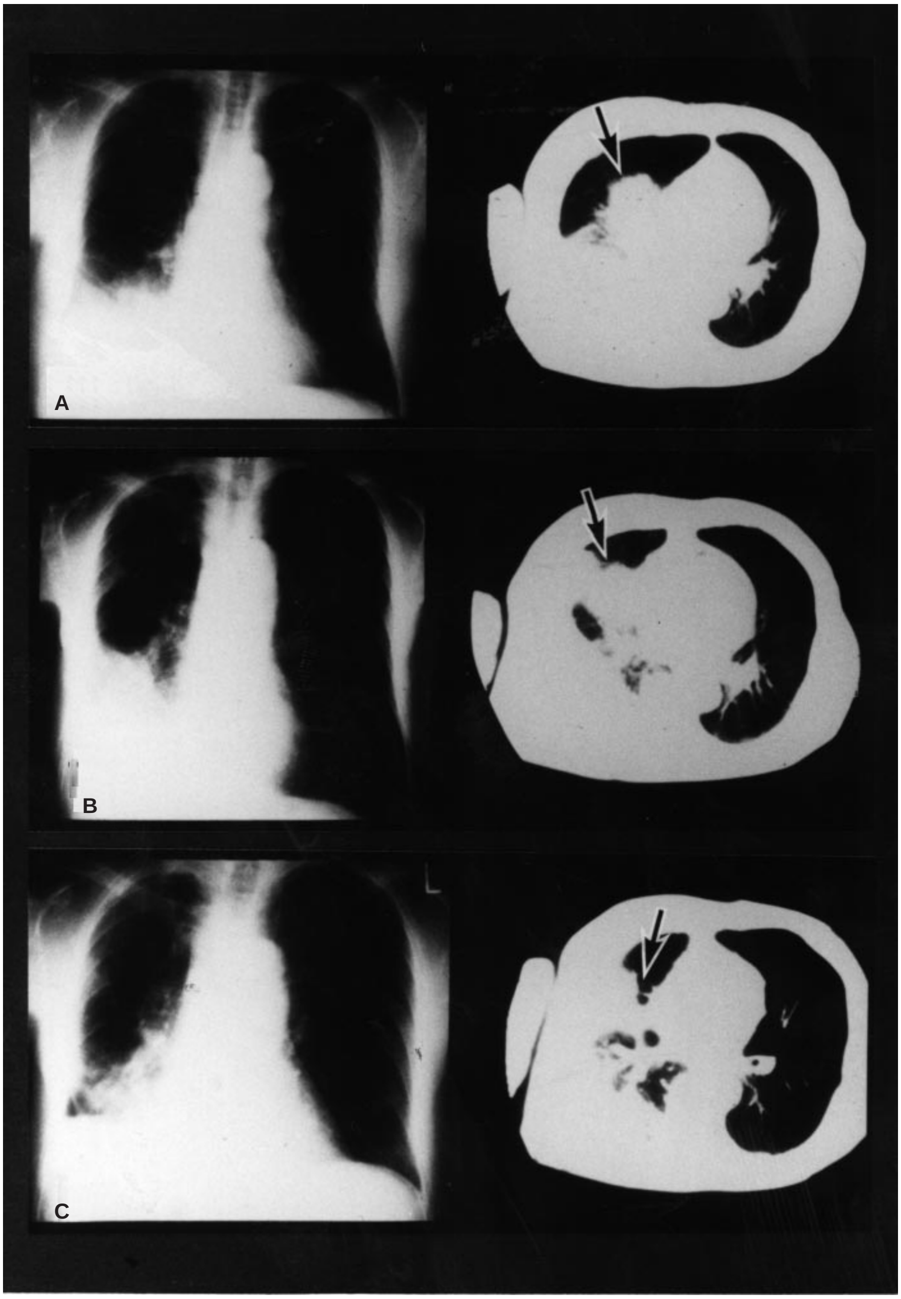

**Figure 3** An 87-year-old male patient with malignant pleural effusion caused by lung cancer. The patient came to our department complaining of dyspnoea, and chest X-ray and CT examinations revealed massive pleural effusion and lung tumour (**A**). Cytologic examination also revealed the malignant cells (adenocarcinoma). He was assigned to Group C. He wanted an ambulatory treatment, and was treated with a weekly puncture of the effusion and an intrapleural injection of EPIR (10 mg $m^{-2}$) and OK-432 (1 KE per body), simultaneously. He received injections a total of 20 times, and the effusion shrunk (MR), but never disappeared (**B**,**C**). Fortunately, he did not suffer from dyspnoea, and lived with a good quality of life for more than 1 year. Finally, he died of disease progression (survival period, 16 months)

imposed by the Japanese national health insurance system, we did find that i.c. immunochemotherapy with OK-432 and ACAs in combination was significantly more beneficial than i.c. chemotherapy alone or immunotherapy alone. The inhomogeneity of chemotherapeutic agent selection for the treatment regimens in Group A and Group C is a major problem in the present study. This problem might induce the results that the lower response rate of objective and cytologic responses in Group A. Furthermore, the possibility that the decrease in Group A's survival rate might be due to higher cytotoxicity of ACAs cannot be excluded. However, this cannot explain the lower rate of objective and cytologic response, and the doses of the agents administered were not so different between the Group A and Group C (Table 3). Accordingly, it seems that the combination of ACAs and OK-432 resulted in the higher rates of objective and cytologic response and better survival. In the present study, the combination of OK-432 and ACAs achieved complete disappearance of the effusion in 43% of patients, and a 17% 1-year survival rate. This combination

 © Cancer Research Campaign 1999

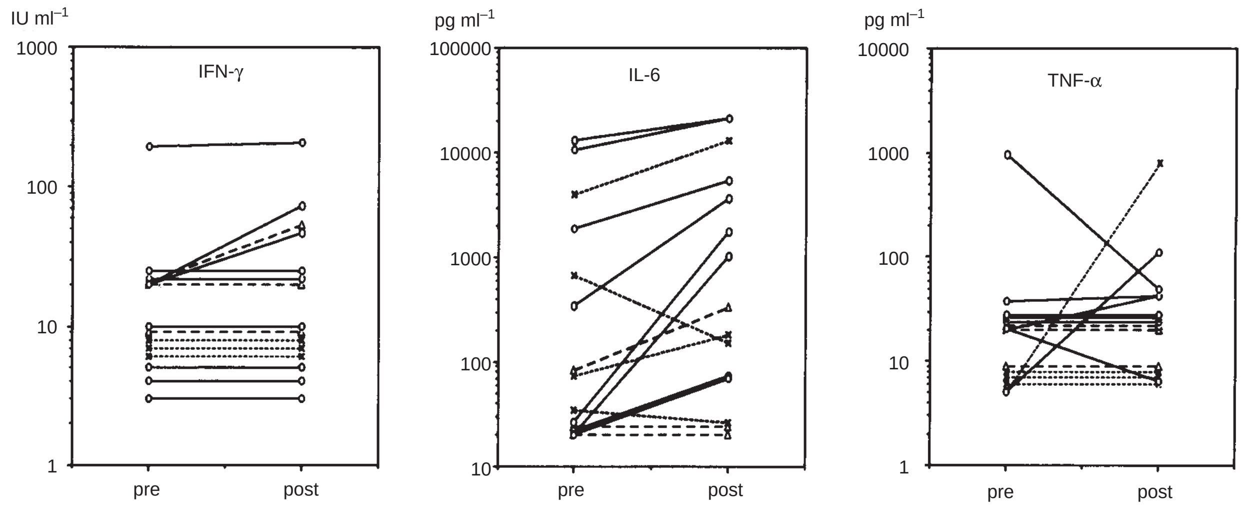

**Figure 4** Cytokines in effusion. x - - - - x, Group A (chemotherapy alone); Δ—Δ, Group B (OK-432 alone); O—O, Group C (chemotherapy + OK-432)

regimen with ACA and OK-432 achieved a higher response rate and survival rate than did ACA or OK-432 alone. Several investigators reported the beneficial effects of combinations of OK-432 and ACAs: the survival rate of patients with lung cancer was higher after treatment with chemotherapy and OK-432 than after treatment without OK-432 (Kimura et al, 1976). The survival of patients receiving immunochemotherapy with intradermal OK-432 and ACAs was significantly better than that of matched control patients given chemotherapy alone (Uchida and Hoshino, 1980). The survival of patients with gastric cancer, who were administered intramuscular OK-432 in addition to the adjuvant chemotherapy after gastrectomy, was significantly better than that of patients who received adjuvant chemotherapy alone (Kim, 1987); a statistically significant improvement of the survival rate after lung cancer surgery was seen in patients treated with OK-432 alone or OK-432 and chemotherapy in comparison with the patients who were treated with chemotherapy alone (Watanabe and Iwa, 1987); and, a post-surgical adjuvant immunochemotherapy with oral OK-432 and MMC + 5-FU derivatives after curative resection of the gastric cancer resulted in a significantly better survival in comparison with chemotherapy alone (Kyoto Research Group for Digestive Organ Surgery, 1992). The mechanisms responsible for the benefits of the combination of OK-432 and chemotherapy are unclear. There are several possibilities: (1) OK-432 augments the anti-tumour effect of ACAs, because OK-432 itself has a direct cytotoxic and cytostatic activity against tumour cells and inhibits DNA and RNA syntheses in tumour cells (Ono et al, 1973; Nio et al, 1990); (2) chemotherapy increases the susceptibility of tumour cells to cytotoxic effecter cells including lymphocytes, macrophages and neutrophils activated by OK-432 through direct damage or modulation of surface antigens by chemotherapy (Ujiie, 1989; Mizutani et al, 1992*b*); (3) ACAs, including ADR and CDDP, directly augment the cytotoxic activity of the effecter cells under certain conditions (Kleinerman et al, 1980; Ehrke et al, 1984), although ACAs usually inhibit the generation of cytotoxic effecter cells (Mantovani et al, 1978; Allavena et al, 1990); and (4) ACAs eliminate the suppressor cells or suppressor factor in the blood or effusion (Heppner and Calabresi, 1972; Goto et al, 1981), resulting in augmented anti-tumour activity of OK-432-activated immunopotentiating cells, especially T-cells (Nio et al, 1989*a*; Bier, 1987; Bier and Bier, 1987). The present immunological study demonstrated that the combination of ACA and OK-432 in Group C resulted in a prominent increase in the level of IL-6 in the effusion. These changes in cytokine levels suggest that immunological responses were caused by the agents. The increase in the level of IL-6 may induce the differentiation and proliferation of various lymphoid cells, especially B-cells, which was seen in all three groups. However, the increase in the level of IL-6 was more prominent in Group C. The increase in the level of IL-6 may be a non-specific inflammatory reaction caused by ACAs and/or OK-432. These results suggest that the immunological reaction might be caused more strongly by the combination of ACAs and OK-432. Accordingly, all of the above mechanisms may be responsible for the combination effects of OK-432 and ACAs.

OK-432 is known to induce a serious adhesion or sclerosis of the peritoneum and pleura when administered into the peritoneal or pleural cavity. Accordingly, i.c. administration of OK-432 has been applied to manage refractory spontaneous pneumothorax. Furthermore, the intracystic injection of OK-432 is reported to be very beneficial for sclerosing therapy of neck cystic hygroma in children (Ogita et al, 1987). These sclerosing effects of OK-432 on the peritoneum and pleura may be derived from the inflammations caused by OK-432. However, the effects sometimes cause serious ileus when OK-432 is administered into the peritoneal cavity. In this study, two patients (one patient in Group B and one patient in Group C) suffered from serious ileus and received total parenteral nutrition although their ascites completely disappeared. The ileus impairs the quality of life (QOL) of the patient, even if the malignant effusion completely disappears. However, the QOL is dependent on the patients' background such as age, social status and previous course of the disease. For example, one of the patients did not voice strong objections to parenteral nutrition and ileus symptoms, since he was pleased to receive relief from effusion and dyspnoea. Because he had suffered from serious abdominal distension and dyspnoea, and sometimes needed oxygen therapy, the

© Cancer Research Campaign 1999

effusion and dyspnoea had also impaired his QOL seriously. However, the other patient suffered from serious vomiting and was continuously drained of gastric and bowel juices. Group C patients enjoyed a survival benefit and a high rate of complete disappearance of effusion. The Group B protocol showed the latter advantage, but no survival benefit, compared with Group A. Balancing the advantages and disadvantages (potential for ileus), the protocol of Group B cannot be justified compared with that of Group A. Accordingly, OK-432 should be administered in combination with ACAs for malignant effusion, and the duration and frequency of i.c. OK-432 administration are key factors for the successful treatment of malignant ascites without adhesion ileus.

In conclusion, the peritoneal or pleural cavity is a restricted space and agents injected i.c. may accumulate in the cavity more easily and efficiently than those injected systemically. Accordingly, immunologic or cytological reactions may be more pronounced after i.c. injection of OK-432 and ACAs than after systemic administration.

### ACKNOWLEDGEMENTS

We gratefully acknowledge Ms Tomoko Toga, Ms Miyuki Ishihara, Ms Yasuko Sonoyama, and Ms Rumiko Hayashi for their technical and secretarial assistance.

### REFERENCES

Allavena P, Pirovano P, Bonazzi C, Colombo N, Mantovani A and D'Incalci M (1990) In vitro and in vivo effects of cisplatin on the generation of lymphokine-activated killer cells. *J Natl Cancer Inst* **82**: 139–142

Bier H (1987) Animal experiments on the role of T lymphocytes in the course of antineoplastic chemotherapy; II. Chemotherapy and T lymphocyte depression. *J Otorhinolaryngeal Relat Spec* **49**: 57–66

Bier H and Bier J (1987) Animal experiments on the role of T lymphocytes in the course of antineoplastic chemotherapy; I. Chemotherapy and tumor-specific immunity. *J Otorhinolaryngeal Relat Spec* **49**: 48–55

Ehrke MJ, Ryoyama K and Cohen SA (1984) Cellular basis for adriamycin-induced augmentation of cell-mediated cytotoxicity in culture. *Cancer Res* **44**: 2497–2504

Fujita K (1987) The role of adjunctive immunotherapy in superficial bladder cancer. *Cancer* **59**: 2027–2030

Fukuma K, Matsuura K, Shibata S, Nakahara K, Fujisaki S and Maeyama M (1986) Pseudomyoma peritonei: effect of chronic continuous immunotherapy with a streptococcal preparation, OK-432 after surgery. *Acta Obstet Gynecol Scand* **65**: 133–137

Gehan EA (1965) A generalized Wilcoxon test for comparing arbitrarily singly-censored sample. *Biometrika* **52**: 203–223

Gehan EA (1975) Statistical methods for survival time studies. In *Cancer Therapy Prognostic Factors and Criteria of Response*. A monograph of the European Organization for Research on Treatment of Cancer. Staquet MJ (ed), pp. 7–35. Raven Press: New York

George SL and Desu MM (1974) Planning the size and duration of a clinical trial studying the time to some critical event. *J Chron Dis* **27**: 15–24

Goto M, Mitsuoka A, Sugiyama M and Kitano M (1981) Enhancement of delayed hypersensitivity reaction with variety of anti-cancer drugs. *J Exp Med* **154**: 204–209

Heppner GH and Calabresi P (1972) Suppression by cytosine arabinoside of serum-blocking factors of cell-mediated immunity to syngeneic transplants of mouse mammary tumors. *J Natl Cancer Inst* **48**: 1161–1167

Inoue K, Kan N, Okino T, Mise K, Moriguchi Y, Nio Y and Tobe T (1993) Surgical management of advanced gastric cancer with peritoneal involvement. *Asian J Surg* **16**: 53–59

Kaplan EL and Meier P (1958) Nonparametric estimation from incomplete observations. *J Am Stat Assoc* **53**: 457–481

Katano M and Torisu M (1982) Neutrophil-mediated tumor cell destruction in cancer ascites. *Cancer* **50**: 62–68

Kawagoe K and Masuda H (1986) Advanced ovarian cancer treated by intraperitoneal immunotherapy with OK-432. *Jpn J Clin Oncol* **6**: 137–142

Kim JP (1987) The concept of immunochemosurgery in gastric cancer. *World J Surg* **11**: 465–472

Kimura I, Ohnishi T, Yasuhara S, Sugiyama M, Urabe Y, Fujii M and Machida K (1976) Immunochemotherapy in human lung cancer using the streptococcal agent OK-432. *Cancer* **37**: 2201–2203

Kitsuki H, Uchiyama A, Yoshida T and Torisu M (1994) OK-432-induced enhancement of ICAM-1 expression on tumor cells positively correlates to therapeutic effects for malignant effusion. *Clin Immunol Immunopathol* **71**: 89–95

Kleinerman ES, Zwelling LA, Schwartz R and Muchmore AV (1980) Defective monocyte killing in patients with malignancies and restoration of function during chemotherapy. *Lancet* **22**: 1102–1105

Kyoto Research Group for Digestive Organ Surgery (1992) A comprehensive multi-institutional study on postoperative adjuvant immunotherapy with oral streptococcal preparation OK-432 for patients after gastric cancer surgery. *Ann Surg* **216**: 44–54

Luh KT, Yang PC, Kuo SH, Chang DB, Yu CJ and Lee LN (1992) Comparison of OK-432 and mitomycin C pleurodesis for malignant pleural effusion caused by lung cancer. *Cancer* **69**: 674–679

Maehara Y, Sugimachi K, Akagi M, Kakegawa T, Shimazu H and Tomita M (1992) Early postoperative chemotherapy following noncurative resection for patients with advanced gastric cancer. *Br J Cancer* **65**: 413–416

Mantovani A, Luini W, Peri G, Vecchi A and Spreafico F (1978) Effect of chemotherapeutic agents on natural cell-mediated cytotoxicity in mice. *J Natl Cancer Inst* **61**: 1255–1261

Miller AB, Hoogstraten B, Staquet M and Winkler A (1981) Reporting results of cancer treatment. *Cancer* **47**: 207–214

Mizutani Y, Nio Y and Yoshida O (1992*a*) The streptococcal preparation OK-432 specifically augments the susceptibility of human urinary bladder tumor cells to autologous peripheral blood lymphocytes. *Cancer* **69**: 2999–3007

Mizutani Y, Nio Y and Yoshida O (1992*b*) Modulation by cis-diamminedichloroplatinum (II) of the susceptibilities of human T24 lined and freshly separated autologous urinary bladder transitional carcinoma cells to peripheral blood lymphocytes and lymphokine activated killer cells. *J Urol* **147**: 505–510

Mukai M, Kubota S, Morita S and Akanuma A (1995) A pilot study of combination therapy of radiation and local administration of OK-432 for esophageal cancer. *Cancer* **75**: 2276–2280

Nio Y, Ohgaki K and Tobe T (1989*a*) Induction by cyclophosphamide administration of two distinct anti-tumor effector cells at tumor site and spleen of mice transplanted with MOPC-104E plasmacytoma. *J Clin Lab Immunol* **29**: 37–43

Nio Y, Zighelboim J, Berek JS and Bonavida B (1989*b*) Cytotoxic and cytostatic effects of the streptococcal preparation OK-432 and its subcellular fractions on human ovarian tumor cells. *Cancer* **64**: 434–441

Nio Y, Zighelboim J, Berek JS and Bonavida B (1990) Sensitivity of ovarian tumor cells to effector cells generated by various biological response modifiers. *Nat Immun Cell Growth Regul* **9**: 283–296

Ogita S, Tsuto T, Tokiwa K and Takahashi T (1987) Intracystic injection of OK-432: a new sclerosing therapy for cystic hygroma in children. *Br J Surg* **74**: 690–691

Ono T, Kurata S, Wakabayashi K, Sugawara Y, Saito M and Ogawa H (1973) Inhibitory effect of a streptococcal preparation (OK-432) on the nucleic acid synthesis in tumor cells in vitro. GANN (*Jpn J Cancer Res*) **64**: 59–69

Osterowski M (1986) An assessment of the long-term results of controlling the reaccumulation of malignant effusions using intracavity bleomycin. *Cancer* **57**: 721–727

Paladine W, Cunningham TJ, Sponzo R, Donovan M, Olson K and Horton J (1976) Intracavitary bleomycin in the management of malignant effusions. *Cancer* **38**: 1903–1908

Peto R, Pike MC, Armitage P, Breslow NE, Cox DR, Howard SV, Mantel N, McPherson K, Peto J and Smith PG (1977) Design and analysis of randomized clinical trials requiring prolonged observation of each patient. *Br J Cancer* **35**: 1–39

Rusch V, Figlin R, Godwin D and Piantadosi S (1991) Intrapleural cisplatin and cytarabine in the management of malignant pleural effusions: a Lung Cancer Study Group trial. *J Clin Oncol* **9**: 313–319

Saito M, Ichimura O, Kataoka M, Moriya Y, Ueno K, Sugawara Y and Nanjo M (1986) Pronounced antitumor effect of LAK-like cells induced in the peritoneal cavity of mice after intraperitoneal injection of OK-432, a killed streptococcal preparation. *Cancer Immunol Immunother* **22**: 161–168

© Cancer Research Campaign 1999

Sakurai Y, Tsukagoshi S, Satoh H, Akiba T, Suzuki S and Takagaki Y (1972) Tumor-inhibitory effect of a streptococcal preparation (NSC-B116209). *Cancer Chemother Rep* **56**: 9–17

Tokai Cooperative Study Group for Adjuvant Chemo-immunotherapy of Stomach Cancer (1981) A controlled study of maintenance chemoimmunotherapy vs immunotherapy alone immediately following palliative gastrectomy and induction chemoimmunotherapy for advanced gastric cancer. *Cancer Chemother Pharmacol* **7**: 5–10

Torisu M, Katano M, Kimura Y, Itoh H and Takesue M (1983) New approach to management of ascites with a streptococcal preparation, OK-432. I. Improvement of host immunity and prolongation of survival. *Surgery* **93**: 357–364

Uchida A and Hoshino T (1980) Clinical studies on cell-mediated immunity in patients with malignant disease. I. Effect of immunotherapy with OK-432 on lymphocyte subpopulation and phytomitogen responsiveness in vitro. *Cancer* **45**: 476–483

Uchida A and Micksche M (1983) Lysis of fresh human tumor cells by autologous peripheral blood lymphocytes and pleural effusion lymphocytes activated by OK432. *J Natl Cancer Inst* **71**: 673–680

Ujiie T (1989) Increased sensitivity of tumor cells to immune defense cells following treatment with antineoplastic agents in vitro. *Jpn J Exp Med* **59**: 17–26

Watanabe Y and Iwa T (1987) Clinical value of immunotherapy with the streptococcal preparation OK-432 in non-small cell lung cancer. *J Biol Res Mod* **6**: 169–180

*© Cancer Research Campaign 1999*
